# Iron Oxidation in *Escherichia coli* Bacterioferritin Ferroxidase Centre, a Site Designed to React Rapidly with H_2_O_2_ but Slowly with O_2_

**DOI:** 10.1002/ange.202015964

**Published:** 2021-03-05

**Authors:** Jacob Pullin, Michael T. Wilson, Martin Clémancey, Geneviève Blondin, Justin M. Bradley, Geoffrey R. Moore, Nick E. Le Brun, Marina Lučić, Jonathan A. R. Worrall, Dimitri A. Svistunenko

**Affiliations:** ^1^ School of Life Sciences University of Essex Wivenhoe Park Colchester Essex CO4 3SQ UK; ^2^ Université Grenoble Alpes CNRS, CEA, IRIG Laboratoire de Chimie et Biologie des Métaux, UMR 5249 17 rue des Martyrs 38000 Grenoble France; ^3^ School of Chemistry University of East Anglia Norwich Research Park Norwich Norfolk NR4 7TJ UK

**Keywords:** EPR spectroscopy, fast kinetics, ferroxidase center, Mössbauer spectroscopy, rapid freeze-quenching

## Abstract

Both O_2_ and H_2_O_2_ can oxidize iron at the ferroxidase center (FC) of *Escherichia coli* bacterioferritin (EcBfr) but mechanistic details of the two reactions need clarification. UV/Vis, EPR, and Mössbauer spectroscopies have been used to follow the reactions when apo‐EcBfr, pre‐loaded anaerobically with Fe^2+^, was exposed to O_2_ or H_2_O_2_. We show that O_2_ binds di‐Fe^2+^ FC reversibly, two Fe^2+^ ions are oxidized in concert and a H_2_O_2_ molecule is formed and released to the solution. This peroxide molecule further oxidizes another di‐Fe^2+^ FC, at a rate circa 1000 faster than O_2_, ensuring an overall 1:4 stoichiometry of iron oxidation by O_2_. Initially formed Fe^3+^ can further react with H_2_O_2_ (producing protein bound radicals) but relaxes within seconds to an H_2_O_2_‐unreactive di‐Fe^3+^ form. The data obtained suggest that the primary role of EcBfr in vivo may be to detoxify H_2_O_2_ rather than sequester iron.

## Graphical abstract

The kinetics of *E. coli* bacterioferritin di‐ferrous ferroxidase centre reacting with O_2_ and H_2_O_2_ shows that H_2_O_2_ reacts circa 1000 times faster than O_2_ implying that the primary in vivo role of the protein is ROS detoxification rather than iron sequestering.

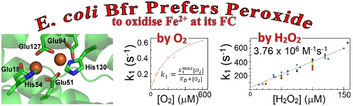

## Introduction

Ferritins belong to the family of proteins and enzymes that exploit the chemistry of dinuclear iron complexes. The di‐iron complexes embedded in proteins have many biochemical functions including catalytic organic transformation (in ribonucleotide reductases,^[1]^ RNR, methane monoxygenases^[2]^ and desaturases^[3]^) as well as reversible O_2_ binding (in haemerythrins,^[4]^ Hr). In addition to these roles, the di‐iron centers in ferritins function as Fe^2+^ oxidases and iron transit sites involved in the formation of polynuclear iron minerals.^[5]^ The oxidation of iron is coupled to reduction of O_2_ (or H_2_O_2_) at the di‐iron centers. This activity has earned them the name *ferroxidase centers* (FC).

Ferritins are typically assemblies of 24 four α‐helix bundles, all or some containing a FC. One ferritin molecule can accommodate thousands of iron atoms in the central mineral core, but iron sequestering, being the primary function for some ferritins^[6]^ is not necessarily the primary in vivo role of all ferritins. Acting as an antioxidant seems to be important for some, particularly in those cases when H_2_O_2_ appears to be the preferred oxidant.^[7]^

For example, the mini‐ferritin Dps (DNA‐binding Protein under Starvation) is a 12meric protein with dinuclear iron complex coordinated with ligands provided by both dimer subunits.^[8]^ Dps utilizes H_2_O_2_ rather than O_2_ and is thought to protect DNA from oxidative damage under conditions of nutritional stress.^[7c]^ This is in contrast to *E. coli* ferritin FtnA which has its primary role in iron homeostasis in metabolically active cells, and the animal H‐chain ferritins—all of which prefer O_2_ as the main co‐substrate for Fe^2+^ oxidation.^[6]^

In this study, we focus on *Escherichia coli* bacterioferritin (EcBfr) for which H_2_O_2_ was reported to compete with O_2_ very successfully in iron oxidation.^[9]^ The rate of EcBfr‐mediated iron oxidation by H_2_O_2_ was estimated to be 10‐fold higher than by O_2_.^[7b]^ We re‐evaluate this factor in this manuscript as a ≈1000‐fold (vide infra). Bacterioferritins (Bfrs) differ from other ferritins in the ligand set of their di‐iron sites^[10]^ but, most importantly, in that they can contain up to 12 haem groups at the two‐fold symmetry binding sites at the interface of two subunits in twelve dimers.^[11]^ The haem is thought to play a role in passing an electron to an iron atom in the core—for it to be reduced and released to the solution.^[12]^ Interestingly, it appears that an electron can also be transferred from reduced Fe^2+^ haem directly to the FCs.^[13]^

**Figure 1:**
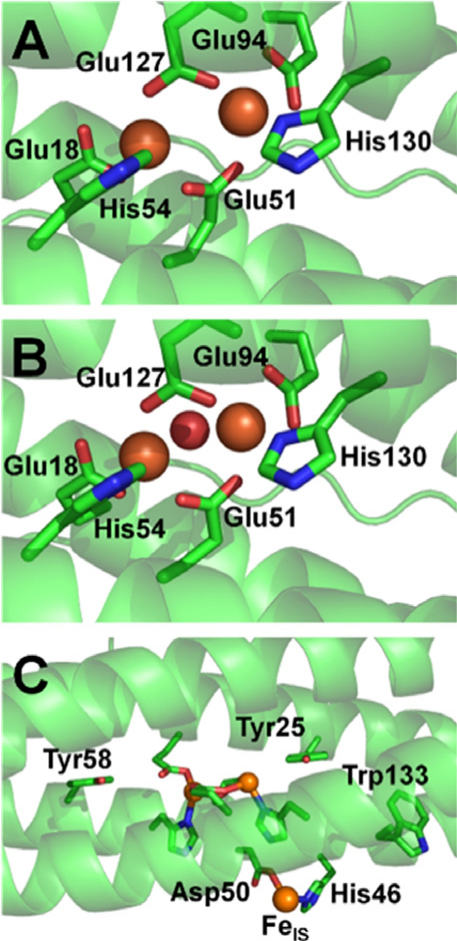
The ferroxidase center (FC) of *Escherichia coli* bacterioferritin (EcBfr) in different oxidation states. A) The EcBfr FC with two Fe^2+^ ions bound (PDB 3E1M^[14b]^). B) Structure with Fe^3+^ ions bound at the FC (PDB 3E1N^[14b]^). The density between the two ferric ions was assigned to the oxygen atom of an oxo‐ or hydroxo‐bridge connecting the two.^[14b]^ C) A zoomed out view of the di‐ferrous structure showing the aromatic residues thought to be involved in mineralisation[^14a^, ^15^] and the IS iron binding site coordinated by Asp50 and His46. This iron is 9.2 Å from the nearest FC iron and 10.2 Å from the second and protrudes into the central cavity. The IS iron is not observed in the di‐ferric structure.^[14b]^.

Figure 1 illustrates the structure of the EcBfr FC when the two iron atoms are in the Fe^2+^ and Fe^3+^ oxidation states. EcBfr also has another iron binding site on the inner surface (IS) of the shell, Fe_IS_. Replacement of the aromatic residues Tyr25, Tyr58 or Trp133, or either of the two residues coordinating the IS iron site (Figure 1 C), significantly affected iron mineralisation.^[14]^ These findings have led to the conclusion that the three aromatic residues and the Fe_IS_ site participate in the electron transfer from the ferrous iron inside the core to the ultimate oxidant (O_2_).[^14^, ^15^]

The FC's ligand arrangement in EcBfr is identical, as far as the first coordination sphere is concerned, with that in *Pseudomonas aeruginosa* BfrB,^[12c]^ a *P. aeruginosa* Bfr (and is similar to ligand sets in RNR[^1^, ^16^] and methane monoxygenase^[2a]^). However, the ligand geometries in these two Bfrs are different enough to result in the very different chemistries these proteins exhibit. We have maintained the view,[^5b^, ^5c^] shared by others,^[17]^ that a common mechanism of mineralisation in ferritins^[18]^ does not exist, and the studies of BfrB support this view. While the FCs of EcBfr are stable and function as pure catalytic sites for O_2_ reduction, the Fe^2+^ oxidation at the structurally similar BfrB FC is followed by translocation of Fe^3+^ to the interior cavity.^[5d]^

The stoichiometry of iron oxidation by O_2_ in EcBfr was reported as 4 Fe^2+^:1 O_2_.^[9]^ This is not a trivial result because one O_2_ molecule is extremely unlikely to oxidise 4 iron ions in 2 different FCs in a concerted reaction—there must be an intermediate(s), likely to be H_2_O_2_. However, attempts to quantitatively detect H_2_O_2_ produced during O_2_‐driven iron oxidations on EcBfr were only partially successful.[^7b^, ^9^] If H_2_O_2_ is produced, some could be lost in side reactions and not in reactions with the FCs, thus affecting the 4:1 overall stoichiometry. Such dissipation of H_2_O_2_, at a level of 38 %, has been reported during iron oxidation by O_2_ in a human heteropolymeric ferritin.^[19]^ To further complicate matters, the Fe^2+^:O_2_ stoichiometry of iron oxidation by the human homo‐24meric ferritin HuHF was reported to be 2:1,^[19]^ not 4:1. Even as recently as in 2019, the stoichiometry of Fe^2+^ oxidation by O_2_ in three different ferritins (two human and one horse) was considered to be as vague as either 2:1 or 4:1.^[20]^

Since most experiments on iron oxidation in ferritins have been performed under oxygenated conditions, when H_2_O_2_ might have been formed as an intermediate and contributed to overall iron oxidation, there is an urgent need to understand fully the precise chemistry through which iron is oxidized by O_2_, and also by H_2_O_2_, and how a di‐ferrous site can utilize one or the other as substrate, but avoid generating poisonous reactive oxygen species. We employed a protocol in which deoxygenated Fe^2+^‐loaded EcBfr is mixed with either oxygenated (to a controlled O_2_ concentration) buffer or deoxygenated buffer containing known H_2_O_2_ concentrations. We used UV/Vis static and stopped‐flow spectrophotometry and an anaerobic Rapid Freeze‐Quench (RFQ) method of making samples (45 ms–1 min) for parallel Electron Paramagnetic Resonance (EPR) and Mössbauer spectroscopic analyses. Thus, this work provides a full account of the stoichiometries and kinetics of EcBfr‐mediated iron oxidation by O_2_ and by H_2_O_2_ and allows a comprehensive mechanism for the activity of the FC to be formulated.

## Results

**Figure 2:**
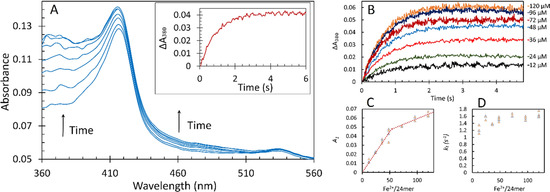
The fast kinetics of 1 μM apo‐EcBfr pre‐loaded anaerobically with Fe^2+^ and reacted with 600 μM O_2_ (in 100 mM MES, pH 6.5, all concentrations are final). A) an example of the photodiode array (PDA) UV/Vis spectra of EcBfr pre‐loaded with 48 μM Fe^2+^ and mixed with O_2_. The spectra correspond to the reaction times: 0.05, 0.30, 0.64, 1.03, 1.53, 2.46 and 19.68 s. The *inset* shows the time dependence of the absorbance at 380 nm increase, Δ*A*_380_, associated with Fe^2+^ to Fe^3+^ oxidation, at a resolution of 3 ms. B) kinetics of Fe^2+^ oxidation to Fe^3+^ (Δ*A*_380_) after addition of 600 μM O_2_ to 1 μM EcBfr pre‐loaded with indicated concentrations of Fe^2+^ (in the mixture). Panels C and D show final absorbance *A*_1_ and pseudo‐first‐order iron oxidation rate constants *k*_1_, respectively, for the seven values of iron loading, obtained from fitting of the kinetic traces (B) with double exponent functions $\Delta A_{380}=\left(A_{1}+A_{2}\right)-{A_{1}e}^{-k_{1}t}-{A_{2}e}^{-k_{2}t}$ . The faster process (*k*_1_) accounts for 90 % of the overall absorbance change observed.

As the protocol employed in our investigations involves incubation of Fe^2+^ anaerobically with the apo‐protein, it is prudent to re‐examine the stoichiometry of Fe^2+^ binding under these conditions for comparison with the earlier approach^[21]^ in which iron was added to aerobic solutions of apo‐protein. The stoichiometries of Fe^2+^ binding to FC under anaerobic conditions (2:1) and of its oxidation thereafter by added O_2_ (4:1) follow from the results reported in Figure 2 and Figure S1.

The amplitude *A_1_* for the rapid phase of iron oxidation increases linearly with [Fe^2+^]—up to approximately 53 Fe^2+^/ EcBfr, which is close to the expected value of 48 for full saturation of the FCs, after which the amplitude continues to increase, but with a shallower slope (Figure 2 C), and does not plateau, as in ref. [21], due to the protocol differences. The first‐order rate constant *k_1_* for this rapid phase is essentially independent of [Fe^2+^] (Figure 2 D) indicating that electron transfer from Fe^2+^ to O_2_ in the 2 Fe^2+^‐O_2_ complex in the FC is slower than O_2_ binding to doubly iron‐occupied FC. The linearity of the titration (Figure 2 C) is consistent with cooperative binding of Fe^2+^ to the FC. Were it otherwise, the fraction of centres with two Fe^2+^ ions bound to FCs, at sub‐stoichiometric [Fe^2+^], would follow a binomial distribution and would not be linear. Cooperative binding of Co^2+^ to the FC has been reported.^[22]^

Consecutive additions of O_2_ saturated buffer aliquots to the (apo‐EcBfr + Fe^2+^)_anaerobic_ system led to progressive oxidation of the Fe^2+^, linearly with [O_2_] until the point of one O_2_ per 4 Fe^2+^ is reached, after which the dependence plateaus (Figure S1). Thus the stoichiometry of iron binding to and oxidation at FCs in the currently employed protocol ((apo‐EcBfr + Fe^2+^)_anaerobic_ + O_2_) is the same as in the protocol used previously ((apo‐EcBfr)_aerobic_ + Fe^2+^).[^9^, ^21^]

**Figure 3:**
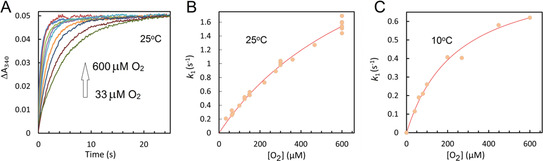
A) The absorbance increase, at 340 nm, as measured by the photomultiplier on addition of buffer with different concentrations of O_2_ to 1 μM EcBfr anaerobically loaded with 48 μM Fe^2+^. All traces were brought to a common endpoint of absorbance change. The data were collected at 25 °C. B) The traces in A, as well as traces obtained in repeats, were fitted to double exponentials and the faster rate constants *k*_1_ (circles) are plotted as function of oxygen concentration. C) the mixing experiments were repeated at 10 °C and *k*_1_ (circles) are plotted as function of oxygen concentration. The data in B and C were fitted to Equation (2) (lines) with parameters reported in Table S1.

Figure 3 reports the kinetics of iron oxidation as a function of O_2_ concentration monitored at 340 nm. The time courses captured at 25 °C (Panel A) were fitted to double exponentials (as in Figure 2, with *A_1_* >90 % of total Δ*A*) and the dependence of *k*_1_ on [O_2_], shown in panel B, is seen to be curved. This suggests O_2_ binds to the Fe^2+^‐loaded FC reversibly and forms an oxy‐complex in which oxidation occurs in a first order process, as shown in Equation 1. This mechanism yields a hyperbolic relationship between *k*_1_ and [O_2_], Equation 2, which we have used to fit the data in Panels B and C. The latter panel shows the data of the experiment repeated at 10 °C (Panel C), where *K*_D_ is expected to be lower and thus the hyperbola more pronounced. Indeed, Figure 3 C shows the hyperbolic nature of the dependence is more obvious, supporting the model of reversible O_2_ binding. The values of $k_{1}^{max}$ and *K*_D_ at 25 °C and 10 °C obtained from the fits of the data to Equation (2) are reported in Table S1.
(1)

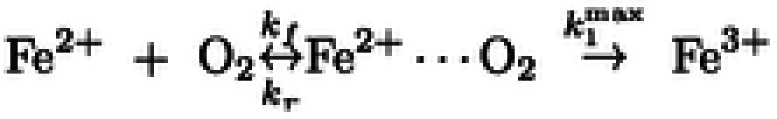

(2) $$k_{1}=\frac{k_{1}^{max}\left[O_{2}\right]}{K_{D}+\left[O_{2}\right]}$$

**Figure 4:**
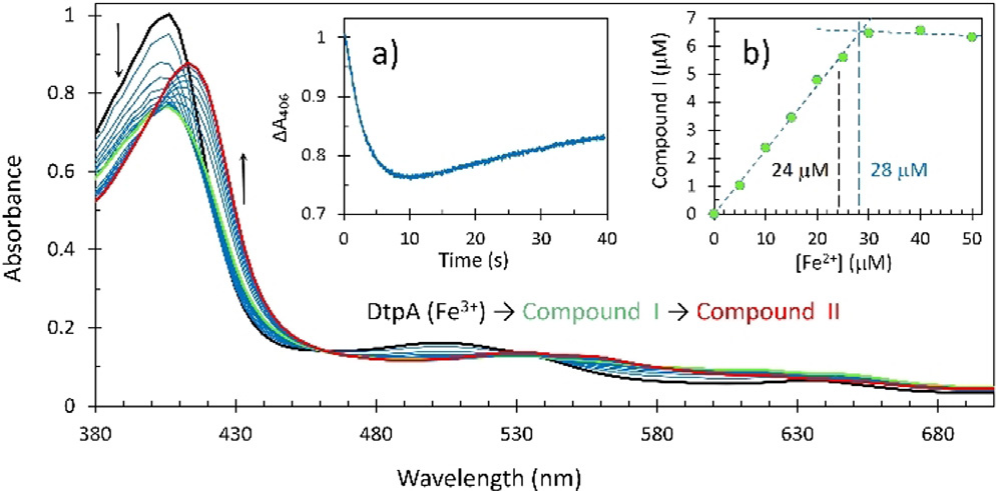
The PDA UV/Vis spectra of 18 μM DtpA in an oxygenated solution of 0.5 μM apo‐EcBfr as it is mixed with 50 μM iron (100 Fe^2+^/24mer). The selected spectra shown are taken at the time points ranging from 40 ms (black spectrum) to 40 s (red spectrum). The spectrum in green corresponds to 9.27 s. *Inset a* shows the absorbance change in the Soret band (at 406 nm) associated with formation of Compound I and its later decay to Compound II. *Inset b* shows formation of Compound I as function of [Fe^2+^] added (see Figure S2B). The first six and the last three data points have been fitted with straight lines, their intercept resulting in [Fe^2+^]=28 μM which is close to the theoretical value of 24 μM of iron load when all FCs are expected to be occupied with iron.

The stoichiometry of 4:1 for iron oxidation with O_2_ makes it very unlikely that four electrons are donated to one O_2_ molecule in a concerted way from four Fe^2+^ ions. It is much more probable that there are steps in the reaction, first of which is oxidation of two Fe^2+^ in the FC to which O_2_ is bound. This would mean that hydrogen peroxide should be formed. If so, does it stay bound to the FC or is it released into solution? To answer this question, we added Fe^2+^ to apo‐EcBfr in air‐equilibrated buffer that contained the dye decolorizing peroxidase DtpA.^[23]^ In the presence of H_2_O_2_, DtpA forms a relatively stable Compound I species, which comprises an oxo‐ferryl haem and a π‐cation radical on the porphyrin,[^23^, ^24^] thus providing a convenient system for H_2_O_2_ detection and quantitation. Figure 4 unambiguously shows that H_2_O_2_ is indeed formed and released to the solution on addition of iron as the DtpA optical spectrum shows changes typical of Compound I formation followed by its decay to Compound II (comprising the same oxo‐ferryl haem but with the radical character now migrated away from the porphyrin). Compound I forms over ca. 5 s (*Inset a*, Figure 4), a time that is consistent with the time course of oxidation of the FC by O_2_ (Figure 3 A). The calculated spectra (Figure S2A) for the DtpA (Fe^3+^) → Compound I → Compound II model are similar to the three highlighted spectra in Figure 4.

To ascertain if all or a part of the H_2_O_2_ formed is released to the solution, stopped‐flow experiments were performed for a range of [Fe^2+^]. Figure S2B shows that the more Fe^2+^ added, the more Compound I is formed. Based on the Δ*ϵ*_390_≈30 000 M^−1^ cm^−1^ (for the absorbance difference $A_{390}^{ferric\;DtpA}-A_{390}^{Compound\;I}$ ) determined from the published spectra,^[24b]^ the concentrations of Compound I at each iron loading have been calculated and are reported in *Inset b*, Figure 4. The yield of Compound I formed in the DtpA+apo‐EcBfr system is directly proportional to the amount of ferrous iron added—up to the concentration required to completely fill all FCs, when Compound I is formed at a high yield of ≈6 μM—a half of a possible maximum of ≈12 μM. This indicates that the second‐order rate constant of FC (doubly filled with Fe^2+^) reacting with H_2_O_2_ must be comparable with that of DtpA reacting with H_2_O_2_ (8.9±0.25×10^6^ M^−1^ s^−1^ at pH 7^[24b]^ and 1.4×10^7^ M^−1^ s^−1^ at pH 6.5 (this work, not shown)).

Thus, H_2_O_2_ is an important player in the FC iron oxidation by O_2_. The stoichiometry of Fe^2+^ to Fe^3+^ oxidation by H_2_O_2_ in the ((apo‐EcBfr + Fe^2+^)_anaerobic_ + H_2_O_2_) system was studied and confirmed to be one peroxide to two Fe^2+^ (Figure S3).

The stopped‐flow PDA UV/Vis spectra of H_2_O_2_ reacting with 48 Fe^2+^/24mer EcBfr are shown in Figure S4A. The noisy time course at 380 nm (*inset*) cannot be used to determine the rate constant of the oxidation accurately. Therefore, we used a photomultiplier that has a much higher time resolution and also can be used at 340 nm, a wavelength used in previous studies.[^15^, ^25^]

**Figure 5:**
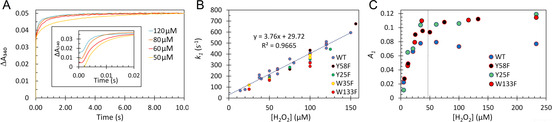
Kinetics of iron oxidation in the FC by H_2_O_2_. A) Four examplary kinetics of absorbance increase at 340 nm on mixing wild type (WT) apo‐EcBfr (1 μM after mixing) anaerobically loaded with Fe^2+^ (48 μM after mixing) with buffer containing H_2_O_2_ (concentrations after mixing indicated). Each trace represents an average of three mixing experiments. All traces were brought to a common endpoint (at 10 s). The *inset* shows the initial 20 ms of the time courses, corresponding to the fast phase of the reaction. B) The values of *k*_1_ obtained from fitting the 340 nm kinetics in the WT and the four EcBfr variants to Equation (3), plotted versus [H_2_O_2_]. C) The amplitude of the fast phase (*A*_1_, see Equation 3) as a function of [H_2_O_2_] for 2 μM apo‐EcBfr anaerobically loaded with 96 μM Fe^2+^]; the stoichiometric [H_2_O_2_] indicated, 48 μM.

Figure 5A shows the time courses of iron oxidation by H_2_O_2_ in the anaerobically prepared Fe^2+^‐EcBfr complex. Those comprise a fast phase and further slower processes—fitted by the triple exponent function given in Equation 3.

(3) $$\Delta A_{340}={(A}_{1}+A_{2}+A_{3})-{A_{1}e}^{-k_{1}t}{{-A}_{2}e}^{-k_{2}t}-{A_{3}e}^{-k_{3}t}$$

The fastest process shows a linear dependence of its pseudo‐first‐order rate constant *k*_1_ on [H_2_O_2_] (Figure 5 B), yielding a second‐order rate constant of 3.76×10^6^ M^−1^ s^−1^. Thus, for comparable concentrations of H_2_O_2_ and O_2_, the rate of iron oxidation by peroxide is ≈1000 times higher than by O_2_ (cf. Figure 5 B & Figure 3 B). Identical data were obtained for four variants (Figure 5 B) in which phenylalanine substituted for aromatic residues previously implicated in iron mineralization.[^14a^, ^15^] The data for the two much slower processes (Figure S5A and Figure S5B) are scattered and will be discussed later.

In Figure 5 C, the amplitude of the fast phase (*A*_1_) of the reaction is shown as a function of [H_2_O_2_], from sub‐ to supra‐stoichiometric concentrations (with a reference to Figure S6), and is seen to increase until sufficient [H_2_O_2_] is present to oxidize all iron in the FCs after which a plateau is reached, showing that H_2_O_2_ is fully consumed in this reaction.

We have previously reported protein radical formation in the (apo‐EcBfr)_aerobic_ + Fe^2+^ system with Tyr25 being the principal site.^[15]^ The rate of Tyr25 radical decay coincides with the rate of a secondary radical(s) formation, and Tyr58 and Trp133 have been shown to be involved in the overall process of radical dissipation.^[14a]^ Having established that H_2_O_2_ reacts with EcBfr anaerobically loaded with Fe^2+^ more than 1000 times faster than O_2_, it is important to determine if H_2_O_2_ leads to protein radicals formation when added to the (apo‐EcBfr + Fe^2+^)_anaerobic_ system. Figure S7 shows that, indeed, free radicals are formed. A comparison of spectra A and B in Figure S7 shows that 250 μM H_2_O_2_ (a slight stoichiometric excess enough to oxidize 250 μM×2=500 μM Fe^2+^ whilst only 400 μM Fe^2+^ are present) yields, a few seconds after mixing, ≈40 times more free radicals than ambient oxygen (sufficient to oxidize 260 μM×4=1040 μM Fe^2+^) when oxidizing the same 500 μM Fe^2+^. Spectra B and C, on the other hand, show that the same concentration of H_2_O_2_ produces far fewer free radicals if it is sub‐stoichiometric to iron—enough to oxidize 500 μM Fe^2+^ whilst the ferrous iron concentration is 1200 μM.

Thus, when O_2_ or H_2_O_2_ oxidizes ferrous ions at FCs, no oxidation equivalents are available to produce free radicals on EcBfr. One way to explain the experimentally observed radicals in O_2_‐ and H_2_O_2_‐treated proteins is to suggest that H_2_O_2_ reacts with di‐ferric FCs. This should result in further oxidation of iron transiently bringing it to a ferryl oxidation state. Its subsequent fast reduction to the ferric state would cause formation of free radicals on protein amino acid residue(s). We now enquire if ferryl iron in the FC can be detected.

**Figure 6:**
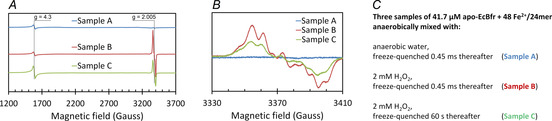
EPR spectra of the RFQ Samples A, B and C prepared as detailed in Experimental Procedures, 1.8, and taken at 23 K. A) overall spectra covering the signals from the rhombic ferric iron at *g*=4.3 and the free radicals at *g*=2.005 (the default instrumental parameters were altered as follows: P_MW_=3.18 mW, Am=5 G, *V*=22.6 G s^−1^). B) detailed free radical EPR region. C) the details of samples A, B and C (see Experimental Procedures, 1.8).

We used a new methodology of making anaerobic RFQ samples for parallel EPR and Mössbauer spectroscopy analysis (Experimental Procedures, 1.7–1.8). The EPR and Mössbauer spectra of the samples are reported in Figure 6 and Figure S8, respectively.

The control Sample A (ferrous as prepared) shows in the EPR spectrum no free radical and a *g*=4.3 EPR signal from rhombic ferric iron which is a sum of the background signal (from the quartz assembly) and residual ferric iron associated with apo‐EcBfr (as prepared at a rather high concentration of the FC, 2 mM). The Mössbauer spectrum of Sample A (Figure S8) exhibits two lines close to −0.3 and +2.9 mm s^−1^ and has been simulated as either one or a superposition of two doublets (Figure S9, Table S2), both cases consistent with high‐spin ferrous ions.[^7d^, ^26^]

A strong free radical EPR signal is recorded in Sample B, while the *g*=4.3 signal is not affected at this time point of the reaction (45 ms, Figure 6). An assessment of the concentration of the free radicals shows it is still a small fraction (≈5–15 %) of the FC concentrations. The very same line shape free radical EPR spectrum but half the intensity is seen 60 s after the reaction starts, and the *g*=4.3 signal is increased (Sample C, Figure 6). Our detailed study of the nature of free radicals formed in EcBfr treated with H_2_O_2_ will be reported elsewhere.

The high velocity line of the ferrous doublet (red dashed line, Figure S8) is not present in the Mössbauer spectra of the H_2_O_2_‐treated samples (B and C) suggesting that all the Fe^2+^ sites are oxidized. The main features in these spectra (Figure S8) are found within the narrow interval of −1 to 2.3 mm s^−1^. To better characterize the species responsible, and to investigate if they are different at 45 ms and 1 min after H_2_O_2_ addition, samples B and C were recorded at a narrower velocity window—±3 mm s^−1^ (at 60 mT) thus providing a better resolution and clear evidence that the iron states differ between 45 ms and 1 min freezing time (Figure S10).

**Figure 7:**
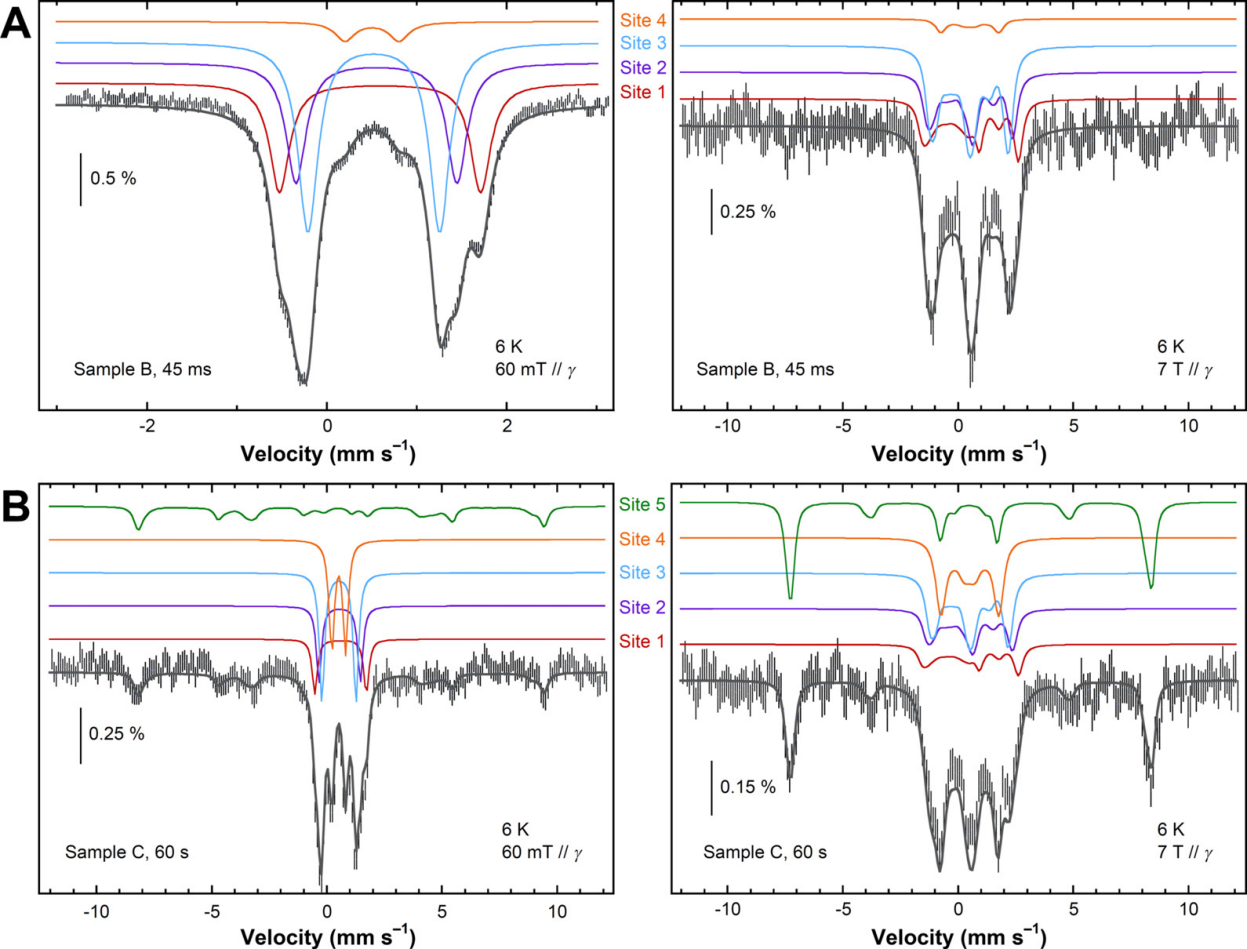
The Mössbauer spectra (hatched bars) of EcBfr treated with H_2_O_2_ and frozen 45 ms (A) and 1 min (B) thereafter (Samples B and C, respectively) measured at 6 K with a 60 mT (left panels) and 7 T (right panels) magnetic field applied parallel to the γ‐rays. The dark grey solid lines are the sums of the simulated contributions displayed as coloured traces above the experimental spectra. Simulation parameters are given in Table S3.

To further investigate these differences, the Mössbauer spectra of Samples B and C were recorded at a greater magnetic field −7 T parallel to the γ‐ray. The spectra of the 45 ms sample measured at 60 mT and 7 T have been simulated as sums of spectra from four *S*=0 iron sites, the diamagnetic character being evidenced by the lack of absorption lines below ≈–2 mm s^−1^ and above ≈+3 mm s^−1^ on the 7 T spectrum (Figure 7 A). The isomer shift values strongly suggest ferric ions that are thus antiferromagnetically coupled to be diamagnetic.

Sample C (frozen 1 min after H_2_O_2_ addition) exhibits 60 mT and 7 T Mössbauer spectra that can be represented as sums of the same four spectra simulated for the 45 ms sample (see Figure 7 A), though in a different combination, plus one more spectrum (Site 5) with the magnetic features spreading over an interval of ≈±9 mm s^−1^ strongly suggesting a *S*=5/2 species (Figure 7 B). The appearance of this paramagnetic ferric species in the Mössbauer spectrum of the 1 min sample is fairly consistent with the increased intensity of the *g*=4.3 signal from the *S*=5/2 species detected by the EPR spectroscopy in the same sample (Figure 6). The correlation between Site 5 content and the concentration of the species responsible for the *g*=4.3 EPR signal is not quantitatively consistent, as far as the data obtained are concerned, and requires further investigation to be statistically confirmed. The intensity of the *g*=4.3 EPR signal is too low for a *g*=9.7 component of the EPR spectrum of high spin Fe^3+^ in rhombic ligand field^[27]^ to be detectable over the noise level—the area covering this g‐value was monitored in the EPR spectra (from 600 G) but showed a flat line and is not included in Figure 6.

None of the simulated lines proposed to contribute to the Mössbauer spectra at 45 ms and 1 min can be linked to a ferryl state. This is in contrast to the previous report of ^57^Fe^4+^ signature in *Pyrococcus furiosus* ferritin (PfFtn), albeit at a low yield of 5±2 % of total ^57^Fe and under O_2_, not H_2_O_2_ treatment.^[28]^

## Discussion

**Figure 8:**
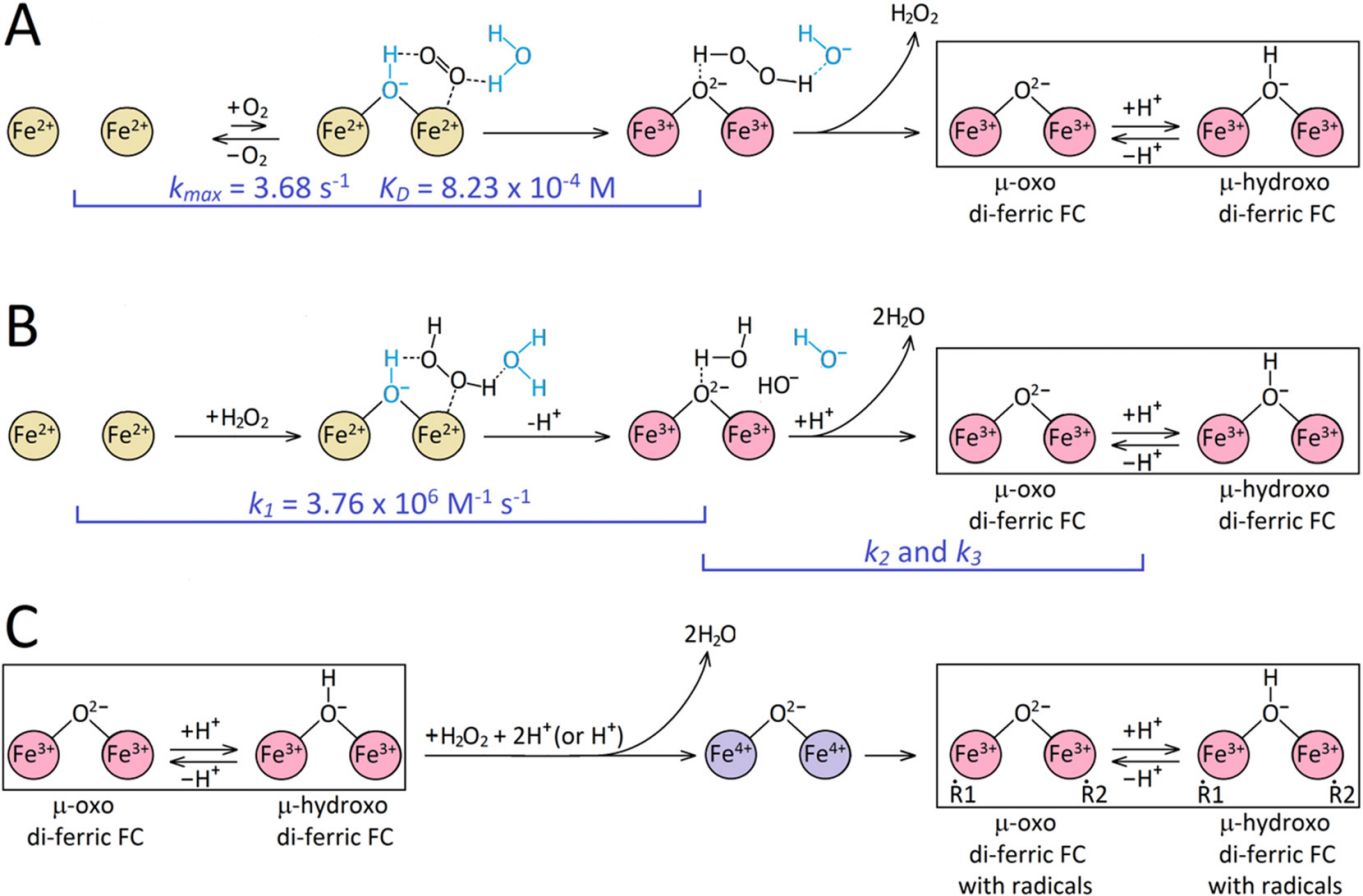
The three sets of reactions of iron oxidation at the FC of EcBfr—by O_2_ (A), by H_2_O_2_ (B) and the reaction of the oxidized (di‐ferric) FC with H_2_O_2_ (C) leading to free radical formation. We put forward the hypothesis that both O_2_ and H_2_O_2_ bind to one of the two iron atoms which must be facilitated by structural water and/or hydroxyls (shown in blue color) or a nearby residue(s), such as Glu94, that provide hydrogen bonding to O_2_ and H_2_O_2_.

The proposed mechanism of iron oxidation at the FC is presented in Figure 8 and comprises three sets of reactions, A, B and C.

**Pathway A** depicts the reactions of a di‐ferrous FC with O_2_. Oxygen binds rapidly but weakly. At ambient [O_2_], taking *K*_D_=823 μM, only ≈24 % of the EcBfr FCs are at any time in the oxy form. At lower [O_2_], likely to be found in the cytoplasm of *E. coli* in its natural environment, the oxy form will be very poorly populated and oxidation of the iron would be extremely slow.

We propose that O_2_ binds to EcBfr in a way similar to Hr—to one of the two iron atoms,^[29]^ Deoxy di‐ferrous Hr has a bridging water (or a hydroxyl group) implicated in such binding^[29]^ but di‐ferrous EcBfr, in contrast, does not show such density in the X‐ray structure^[14b]^ We think, however, it is possible that the cluster of three water molecules near the FC iron coordinated by His130, plus a water molecule close to the other iron of the FC (see the PDB file in the Supporting Information), may be rearranged by in‐coming O_2_ to form a bridge between the two iron atoms as hypothesized in Figure 8. The Hr type O_2_ binding to the di‐ferrous center[^29^, ^30^] has been subjected to theoretical modelling and has been shown to account well for reversible O_2_ binding in Hr.^[31]^ EcBfr, we suggest, may be considered qualitatively similar to Hr but quantitatively different, having a higher *K*_D_ and a much larger “autoxidation” rate constant. An alternative mode of O_2_ binding, in which O_2_ bridges between the iron atoms, leads to rapid electron transfer yielding a peroxo‐bridged di‐ferric center from which O_2_ cannot dissociate (see, for example ref. [32]).

Two electrons transferred from the di‐ferrous FC to O_2_ yield an H_2_O_2_ molecule, which is released to solution (Figure 4). Its reaction path with another FC is given in Figure 8 B.

**Pathway B**. The H_2_O_2_ binding is rapid and proposed to be to one iron, similar to O_2_ binding, with stabilizing hydrogen bonds provided by the water cluster.

Iron oxidation by H_2_O_2_ is much faster than by O_2_. Although noisy, the spectra in Figure S4A and the way they are changing in time are very similar to those in Fe^2+^ oxidation by oxygen (Figure 2 A) and in titration of ferrous EcBfr with either O_2_ (Figure S1) or H_2_O_2_ (Figure S3). The Singular Value Decomposition^[33]^ (SVD) analysis of the complete PDA spectral set also yields two spectral components consistent with the EcBfr(Fe^2+^) to EcBfr(Fe^3+^) transition (Figure S4B). This means that O_2_ and H_2_O_2_ driven oxidation of iron in the FC, while being three orders of magnitude different in rate, produce essentially the same spectral changes both at the earliest stage of reaction and minutes later, for the final products of oxidation.

The second‐order rate constant of iron oxidation by H_2_O_2_, 3.76×10^6^ M^−1^ s^−1^ (Figure 8 B, Figure 5 B), is only ≈2 fold lower than the constant of peroxide reacting with DtpA, a peroxidase for which H_2_O_2_ is a designated substrate, *k*=8.9±0.25×10^6^ M^−1^ s^−1^.^[24b]^ We emphasize that it is this very high rate constant than warrants the 4:1 stoichiometry of iron oxidation by O_2_ (Figure S1)—every molecule of H_2_O_2_ formed in one FC oxidation by an O_2_ is used to oxidize ferrous iron in other FCs.

Interestingly, replacement, with phenylalanine, of the aromatic residues (Figure 5, Figure S5) implicated in iron mineralization by EcBfr,[^14a^, ^15^] had no effect on the rate constants of iron oxidation. Therefore, H_2_O_2_ binding to di‐ferrous FC and its oxidation to the di‐ferric state is unlikely to involve any redox chemistry of the aromatic residues surrounding the FC.

Kinetics of iron oxidation by sub‐ and supra‐stoichiometric [H_2_O_2_] show that the amplitude (*A*_1_) of the fastest process (*k*_1_) is directly proportional to [H_2_O_2_] up to the value required for oxidation of all iron bound to the FCs (Figure 5 C). This result allows the conclusion that all iron is oxidized in the first, fastest phase of absorbance change (ca. 20 ms). As the phase associated with *k*_2_ does not appear until [H_2_O_2_] is in excess (Figure S6), we may assign this process to a second‐order reaction of the excess peroxide with the di‐ferric centers generated in the first, fast, process (see Pathway C below). Further, *k*_3_ has no discernible dependence on [H_2_O_2_] and is present at both sub‐ and supra‐stoichiometric [H_2_O_2_]. This process therefore cannot be associated with electron transfer (oxidation/reduction)—it is much slower than processes 1 and 2 and the only reasonable explanation for it is that it is associated with some structural changes in molecular arrangement. The time scale of these changes is consistent with the process that takes place in the time span 45 ms–1 min as observed in the Mössbauer spectroscopy experiments. We therefore assign this phase to the configurational changes of the FC following its oxidation to the di‐ferric state.

Our conclusions that all iron is oxidized during the fastest phase and that the slower two phases are associated with configurational changes and side reactions with excess H_2_O_2_ are supported by the Mössbauer spectroscopy data.

Without H_2_O_2_, most of ^57^Fe remains in the ferrous state (Figure S9). The EcBfr samples freeze‐quenched 45 ms and 1 min after H_2_O_2_ addition show no ferrous iron remained in the FC (Figure S8). Neither ferryl species are found (Figure 7) which should have significantly smaller values of the isomer shift^[34]^ than those used to simulate the five spectra for the Sites 1–5 (Table S3). On the contrary, the simulation parameters of all five sites identified in the H_2_O_2_‐treated samples are consistent with ferric species.^[35]^

All four iron sites identified in the 45 ms sample are diamagnetic. This means that, at this time point, two ferric ions in every FC remain antiferromagnetically coupled. The contributions of Sites 1 and 2 are almost identical (27–29 %, Table S3), suggesting that these sites belong to the same FC (*dissymmetrical* FC). Their isomer shifts (Table S3) are at the higher limit of the range for ferric ions. This is usually observed for peroxodiferric intermediates.[^34^, ^36^] Two ferric ions (also coupled) in the *symmetrical* di‐ferric FC (giving identical Mössbauer signatures—Site 3, Figure 7, Figure S11) contribute most to the overall spectrum (≈46 %, Table S3). We propose that the two di‐ferric FCs, dissymmetrical (Site 1—Site 2) and symmetrical (Site 3—Site 3), differ in immediate coordination of one of the iron ions. We propose that the (Site 1—Site 2) FC is a peroxodiferric FC in which the peroxo group is bound to one of the two iron ions whereas the two are linked with a μ‐oxo bridge, similarly to the peroxodiferric center in Hr.[^29^, ^34^] The (Site 3—Site 3) FC, on the other hand, does not have this peroxo ligand to one of the ions and shows typical^[35a]^ μ‐oxo di‐ferric (symmetrical) Mössbauer parameters (Figure S11).

All four sites in the 45 ms sample are found in changed proportions in the 1 min sample—Sites 1–3 decrease while Site 4 contribution increases from 5 % to 23 % (Table S3). The isomer shift of Site 4 is close to those of the μ‐oxo species, but its quadrupole splitting is significantly lower which is consistent with a di‐ferric μ‐hydroxo species.[^35a^, ^35c^, ^37^] We propose therefore that Site 4 is formed via protonation of Site 3 (μ‐oxo di‐ferric to μ‐hydroxo di‐ferric Figure S11).

Along with Site 4, another species emerges over the 45 ms −1 min interval—a paramagnetic Site 5 with well‐defined parameters of a high‐spin (*S*=5/2) monomeric iron site. This is evidenced by both the 60 mT and 7 T Mössbauer experiments (Figure 7 B) and supported by the increased *g*=4.3 EPR signal at 1 min (Figure 6).

In reaction set C (**Pathway C,** Figure 8), we propose a mechanism for free radical formation on EcBfr—it can only be explained by the reaction of H_2_O_2_ with oxidized FC already formed. A likely possibility is that one H_2_O_2_ molecule binds to a “freshly” oxidized FC and takes two electrons, in a rapid succession or in concert, from the two ferric ions, thus forming a di‐ferryl (2 Fe^4+^) state. The two ferryl ions are re‐reduced by two different protein residues, thus forming two different protein‐based radicals and returning the FC to the di‐ferric state. (We will report elsewhere that indeed more than one primary radical is formed on EcBfr under excess of H_2_O_2_). These redox processes, and the conformation/coordination changes that follow, take place over a much longer time scale than primary Fe^2+^→Fe^3+^ oxidation and must be associated with the slower kinetic phases (with rate constants *k*_2_ and *k*_3_) of the absorbance increase (Figure S5, Figure S6).

The need to postulate a “freshly” oxidized FC follows from the experimental fact that H_2_O_2_ does not produce any radical if added to an EcBfr sample fully loaded with iron and oxidized to a di‐ferric state a few minutes earlier. This means that the “relaxed” oxidized FC cannot react with H_2_O_2_, while just oxidized but not “relaxed” FC can. In terms of the iron sites identified from the Mössbauer spectra, the “freshly” oxidized FC are Sites 1, 2 and 3 (all three seen in the 45 ms sample) and the “relaxed” oxidized FC is associated with sites 4 and 5 (elevated over 45 ms–1 min, while sites 1, 2 and 3 decreased).

Thus, we propose that μ‐oxo bridged di‐ferric state forms first and then is protonated (Figure S11). This hypothesis requires further investigation. It is likely that once the μ‐hydroxo state is formed, further re‐arrangements of the ligands can occur, leading to two unbridged ferric atoms which are now uncoupled, showing paramagnetism and also unavailable for reacting with H_2_O_2_ (Figure S11).

## Conclusion

1. Oxygen binds reversibly and weakly (*K*_D_=823 μM) to the di‐Fe^2+^ site to form an oxy‐complex in which electron transfer takes place, forming H_2_O_2_ that dissociates rapidly and fully into solution.
2. Released peroxide reacts very rapidly (*k*=3.76×10^6^ M^−1^ s^−1^) and quantitatively with remaining di‐Fe^2+^ sites accounting for the 2 Fe^2+^:1 H_2_O_2_ and the 4 Fe^2+^:1 O_2_ stoichiometries.
3. Both oxidizing equivalents of peroxide are delivered to the di‐Fe^2+^ site in the ms time range converting it to the μ‐oxo di‐Fe^3+^ form. No radicals can be formed in this oxidation process. Over tens of seconds, it evolves into protonated, μ‐hydroxo di‐Fe^3+^ form.
4. Excess peroxide reacts with di‐Fe^3+^, to yield protein‐based radicals. We propose a hypothesis that that H_2_O_2_ reacts only with the μ‐oxo and not the μ‐hydroxo bridged di‐ferric ions.

This mechanism shows that at low oxygen concentrations, as may be experienced by *E. coli* in its natural environment, the di‐ferrous iron in the FC is oxidized extremely slowly by O_2_ while oxidation by H_2_O_2_ is at least 1000‐fold faster. This supports the suggestion that one role of EcBfr may be to act as part of an antioxidant defense system, rapidly sequestering and rendering harmless peroxide in the cellular environment.

## Conflict of interest

The authors declare no conflict of interest.

## Supporting information

As a service to our authors and readers, this journal provides supporting information supplied by the authors. Such materials are peer reviewed and may be re‐organized for online delivery, but are not copy‐edited or typeset. Technical support issues arising from supporting information (other than missing files) should be addressed to the authors.

Supplementary

Supplementary
