# Supplementary material for: Iron Oxidation in Escherichia coli Bacterioferritin Ferroxidase Centre, a Site Designed to React Rapidly with H2O2 but Slowly with O2
Source: Angew Chem Weinheim Bergstr Ger. 2021 Mar 5;133(15):8442–50. doi: 10.1002/ange.202015964 (PMC10962548; doi:10.1002/ange.202015964)
Supplement: Supplementary file 2 — Supplementary [file ANGE-133-8442-s001.pdf]

Angewandte Chemie
Eine Zeitschrift der Gesellschaft Deutscher Chemiker

Supporting Information

# Iron Oxidation in *Escherichia coli* Bacterioferritin Ferroxidase Centre, a Site Designed to React Rapidly with $H_2O_2$ but Slowly with $O_2$

*Jacob Pullin, Michael T. Wilson, Martin Clémancey, Geneviève Blondin, Justin M. Bradley, Geoffrey R. Moore, Nick E. Le Brun, Marina Lučić, Jonathan A. R. Worrall, and Dimitri A. Svistunenko**

ange_202015964_sm_miscellaneous_information.pdf
ange_202015964_sm_miscellaneous_information.pdb

Supporting Information
©Wiley-VCH 2019
69451 Weinheim, Germany

# Iron oxidation in *Escherichia coli* bacterioferritin ferroxidase centre, a site designed to react rapidly with $H_2O_2$ but slowly with $O_2$

Jacob Pullin, Michael T. Wilson, Martin Clémancey, Geneviève Blondin, Justin M. Bradley, Geoffrey R. Moore, Nick E. Le Brun, Marina Lučić, Jonathan A. R. Worrall, Dimitri A. Svistunenko*

**Abstract:** Both $O_2$ and $H_2O_2$ can oxidise iron at the ferroxidase centre (FC) of *Escherichia coli* bacterioferritin (EcBfr) but kinetic details of the two reactions are unclear due to $H_2O_2$ being an intermediate of iron oxidation by $O_2$. UV-vis, EPR and Mössbauer spectroscopies were applied to follow the reactions when a protocol was used in which $O_2$ or $H_2O_2$ was added to apo-EcBfr pre-loaded anaerobically with $Fe^{2+}$. We show that $O_2$ binds di-$Fe^{2+}$ FC reversibly, two $Fe^{2+}$ ions are oxidised in concert and a $H_2O_2$ molecule is formed and released to solution. This peroxide molecule further oxidises another di-$Fe^{2+}$ FC, at a rate ~1000 faster than $O_2$, ensuring an overall 1:4 stoichiometry of iron oxidation by $O_2$. Initially formed $Fe^{3+}$ can still react with $H_2O_2$ (producing protein bound radicals) but relaxes within seconds to an $H_2O_2$-unreactive di-$Fe^{3+}$ form. The data obtained suggest that the primary role of EcBfr *in vivo* is rather $H_2O_2$ detoxification than iron sequestering.

**DOI: 10.1002/anie.2016XXXXX**

## Table of Contents

1. Experimental Procedures .page 2
1.1 Over-expression and purification of EcBfr and variants . page 2
1.2. Anaerobic buffers and $O_2$ concentration controlled solutions . page 2
1.3. Spectrophotometric titrations of EcBfr with $H_2O_2$ or $O_2$ page 2
1.4. Stopped-flow UV-vis measurements . page 3
1.5. Slow-freeze method of making samples for EPR spectroscopy . page 3
1.6. EPR spectroscopy . page 3
1.7. Anaerobic Rapid Freeze-Quenching . page 3
1.8. RFQ samples of the ((EcBfr-$^{57}Fe^{2+}$) + $H_2O_2$)$_{anaerobic}$ system for parallel EPR and Mössbauer spectroscopies . .page 3
1.9. Mössbauer spectroscopy .page 4
Supporting figures . page 4
Figure S1 . page 4
Figure S2 . page 5
Figure S3 . page 5
Figure S4 page 6
Figure S5 . page 6
Figure S6 . page 6
Figure S7 page 7
Figure S8 . page 7
Figure S9 . page 8
Figure S10 . page 8
Figure S11 . page 9
Supporting Tables page 9
Table S1 .page 9
Table S2 page 9
Table S3 page 10
Abbreviations and annotations used page 10
References page 10
Author contributions page 11

## 1. Experimental Procedures

### 1.1. Over-expression and purification of EcBfr and variants

Wild type and variant EcBfr proteins were prepared as previously described,[1] with expression induced with 25 µM IPTG. Sodium dithionite and bipyridyl were used to remove non-haem iron.[2] The non-haem iron content in the protein after the procedure has been assessed previously and found to be low (~1 iron per 24mer).[3] The assessment of selected samples in this study gave a range of iron content of 4-10 iron per 24mer or 16-40% of monomer concentration. From the titration of apo-EcBfr with $Fe^{2+}$, it follows that most of this residual ferric iron is not in the ferroxidase centre (FC), and from the intensity of the g = 4.3 EPR signal in the apo-EcBfr (which usually corresponds to 0.5-1% of monomer concentration), it follows that most of the residual ferric iron is antiferromagnetically coupled. The monomeric concentrations of EcBfr were determined using the following $\varepsilon_{280}$ values: 33 000 (WT),[4] 25 585 (Y25F),[1a] 24 600 (Y58F),[1a] 23 375 (W133F),[1b] 22 300 (W35F)[1a] all in units of $M^{-1}cm^{-1}$. The concentration of haem iron was determined using a $\varepsilon_{418}$ value of 107 000 $M^{-1}cm^{-1}$.[5] after non-haem iron removal. All variants were found to contain 0.3-1.5 haem/EcBfr.

### 1.2. Anaerobic buffers and $O_2$ concentration controlled solutions

A Schlenk line was used to prepare anaerobic protein and buffer solutions (100 mM MES, pH 6.5) under argon. Saturated $O_2$ solutions were prepared by bubbling $O_2$ through buffer; the concentrations of $O_2$ in these solutions were calculated as the maximum $O_2$ solubility for the room temperature and the local atmospheric pressure[6] on the day of experiment. This was typically around 1.2 mM $O_2$.

### 1.3. Spectrophotometric titrations of EcBfr with $H_2O_2$ or $O_2$

Degassed proteins in sealed cuvettes were loaded with degassed ferrous ammonium sulfate solution in 50 mM HCl and then titrated with $H_2O_2$ solution in degassed buffer or $O_2$-saturated buffer (see above). UV-vis spectra were recorded more than 1 min after each incremental addition, when any consecutively measured spectra became identical, on a Cary UV-vis spectrophotometer (Agilent Technologies). Incremental dilution during the titration was accounted for in presenting the spectra and the analysis.

### 1.4. Stopped-flow UV-vis measurements

Transient kinetics of the reactions were monitored using an Applied Photophysics SX20 stopped-flow spectrometer equipped with a thermostat (used at 25ºC and 10ºC) and either a photodiode array (PDA) multi-wavelength unit or a photomultiplier. The protein solutions were prepared anaerobically on a Schlenk line before being incubated with $Fe^{2+}$ (see above). Iron loaded EcBfr and anaerobically prepared buffer with either $H_2O_2$ or $O_2$ were transferred carefully to gas-tight syringes which were fitted to the stopped-flow apparatus. Experimental kinetic traces were analysed individually and globally using the Pro-K software (Applied Photophysics).

### 1.5. Slow-freeze method of making samples for EPR spectroscopy

To achieve reaction time (before freezing) from ~10 s and longer (slow-freeze), EcBfr solutions were placed in selected Wilmad SQ EPR tubes (Wilmad Glass, Buena, NJ) with OD = 4.05±0.07 mm and ID = 3.12 ± 0.04 mm (mean ± range). In making (apo-EcBfr$_{aerobic}$ + $Fe^{2+}$) samples, 5 µl of $FeCl_2$ in HCl was delivered using a long needle to the pre-dispensed 250 µl of apo-EcBfr at ambient oxygen. The tubes were then frozen in methanol kept on solid $CO_2$. In making (apo-EcBfr + $Fe^{2+}$)$_{anaerobic}$ + $H_2O_2$ samples, 250 µl of degassed EcBfr solutions, pre-loaded with iron anaerobically, were placed to the bottom of an EPR tube carefully filled beforehand with argon. Hydrogen peroxide solution was then delivered to the protein using plastic tubing, and the mixture was frozen in methanol kept on solid $CO_2$.

### 1.6. EPR spectroscopy

Low temperature EPR spectra were recorded on a Bruker EMX (X-band) EPR spectrometer with the use of an Oxford Instruments liquid-helium system and a spherical high-quality ER 4122 (SP 9703) Bruker resonator. Free radical concentrations were assessed by using a 100 µM $Cu^{2+}$ concentration standard. The instrumental conditions of EPR spectra measurements were as follows, if not stated otherwise: microwave frequency $\nu_{MW}$ = 9.47 GHz, microwave power $P_{MW}$ = 0.05 mW, modulation frequency $\nu_m$ = 100 kHz, modulation amplitude $A_m$ = 3 G, time constant $\tau$ = 82 ms, scan rate V = 0.60 G/s, number of scans per spectrum NS = 1.

### 1.7. Anaerobic Rapid Freeze-Quenching

Rapid Freeze-Quenched (RFQ) EPR samples were prepared on an isopentane-free apparatus as described before.[7] To perform freeze-quenching of reaction mixtures under anaerobic conditions, a glove bag was attached to the apparatus, filled with continuously flowing argon and kept for ~30 min while the RFQ syringes were equilibrated with argon by pushing the plungers up and down several times to remove air. Anaerobic solutions in airtight glass syringes where then brought inside the bag, incubated there for additional 30 min and used to fill the RFQ syringes.

### 1.8. RFQ samples of the ((EcBfr-$^{57}Fe^{2+}$) + $H_2O_2$)$_{anaerobic}$ system for parallel EPR and Mössbauer spectroscopies

The $^{57}$Fe-containing $(NH_4)_2Fe(SO_4)_2$ was prepared from powdered $^{57}$Fe (Cambridge Isotope Laboratories, Inc.) as follows. 57 mg of powdered metallic $^{57}$Fe was dissolved in 500 µl of 2 M $H_2SO_{4(aq)}$ and 150 mg of $(NH_4)_2SO_4$ dissolved in 130 µl of water. Both solutions were heated to 95ºC before combining to generate an aqueous solution of $(NH_4)_2Fe(SO_4)_2$. The sample was crystallised by cooling for 15 mins in a ice/water bath before isolating by vacuum filtration. The crude product was purified by re-crystallising from boiling water. The dry mass of re-crystallised product was 242 mg which gives a yield of 62% based on Fe. Ferritin activity assessed by the assays using the synthesized $(NH_4)_2{}^{57}Fe(SO_4)_2$ was indistinguishable from that assessed using $(NH_4)_2Fe(SO_4)_2$ of 99% purity (Sigma).

A volume of anaerobic 83.3 µM WT apo-EcBfr (3 ml) was mixed inside the glove bag with an aliquot of HCl solution of $^{57}Fe^{2+}$, sufficient to fill all FCs. This mixture was then loaded into one of three Ar-equilibrated syringes of the RFQ apparatus; anaerobic water and a freshly prepared solution of 4 mM $H_2O_2$ dissolved in anaerobic water were simultaneously loaded into two other syringes. Protein samples were mixed with water or $H_2O_2$ and the mixtures were sprayed specific time thereafter onto a rotating aluminium disk thermostatically equilibrated with liquid nitrogen. The longer reaction time (60 s) samples was made using a double-push regime when an aging hose long enough to accommodate a whole one shot volume (the 400 ms hose, 350 µl to fill it) was filled with the reaction mixture, then, after a delay of 59.6 s, the second push expelled the aged volume onto the cold disk of the RFQ apparatus. More than

one shots, at identical RFQ settings, were performed to increase the total amount of RFQ ice to be shared between the EPR and Mössbauer samples.

The following three anaerobic samples have been prepared (indicated concentrations are in the mixtures):

Sample A - 41.7 µM apo-EcBfr + 48 $Fe^{2+}$/24mer + anaerobic water 3 shots
Sample B - 41.7 µM apo-EcBfr + 48 $Fe^{2+}$/24mer + 2 mM $H_2O_2$ + 45 ms 3 shots
Sample C - 41.7 µM apo-EcBfr + 48 $Fe^{2+}$/24mer + 2 mM $H_2O_2$ + 60 s 2 shots

The 2 mM $H_2O_2$ is sufficient to oxidise 4 mM of $Fe^{2+}$ while the total concentration of $^{57}Fe^{2+}$ in each sample is 2 mM (41.7 µM x 48).

The frozen mixtures were then packed into EPR tubes and Mössbauer cups for parallel assessment by the two spectroscopies.

### 1.9. Mössbauer spectroscopy

The Mössbauer spectra were recorded on samples contained in Delrin cups at 4.2 K on a low-field Mössbauer spectrometer equipped with a Janis SVT-400 cryostat or at ca. 5 K on a strong-field Mössbauer spectrometer equipped with an Oxford Instruments Spectromag 4000 cryostat containing an 8 T split-pair superconducting magnet. Both spectrometers were operated in a constant acceleration mode in transmission geometry. The isomer shifts were referenced against that of a room-temperature metallic iron foil. Analysis of the data was performed with a home-made program.[8]

## Supporting figures

Figure S1

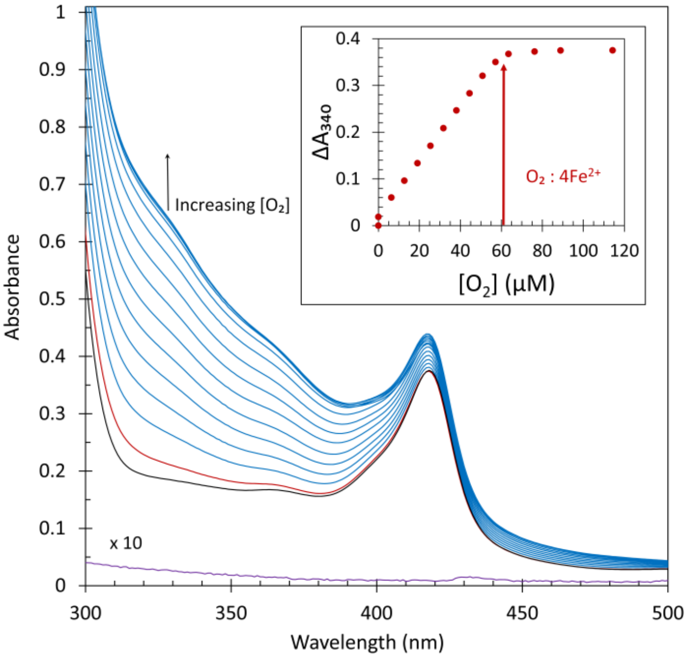


**Figure S1.** UV-vis spectra of the EcBFR-$Fe^{2+}$ complex titrated with oxygen. Apo-EcBFR (5.13 µM) was incubated anaerobically with 246 µM $Fe^{2+}$ (which is 5.13 µM x 24 subunits per EcBFR x 2 iron binding sites per FC) and then MES buffer (100 mM, pH 6.5) saturated with $O_2$ (1.27 mM) was titrated into the mixture. The spectrum of the apo-EcBFR is in black. After $Fe^{2+}$ was added, a small increase in the absorbance observed in the UV region indicates some iron oxidation and must be associated with residual $O_2$ in the system (either in the apo-EcBFR, or in the $Fe^{2+}$ solution or in both) as it cannot be accounted for by the $Fe^{2+}$ contribution to the spectrum because the control spectrum of a buffer solution with 200 µM $Fe^{2+}$, multiplied by a factor of 10 (purple trace at the bottom of the Figure), is of a very low intensity. The blue spectra represent subsequent additions of aliquots of $O_2$-saturated buffer. The *inset* displays the $\Delta A_{340}$ values, corrected for dilution of the protein throughout the titration, as function of [$O_2$]. The red arrow indicates the expected $O_2$ concentration corresponding to the 4:1 stoichiometry of iron oxidation by oxygen ($Fe^{2+}$ : $O_2$). The two data points for the [$O_2$] = 0 correspond to the apo-EcBFR (black trace) and EcBFR loaded with $Fe^{2+}$, before $O_2$ buffer aliquots were added (red trace); this corresponds to 4.6 % of total iron oxidised. .

Figure S2

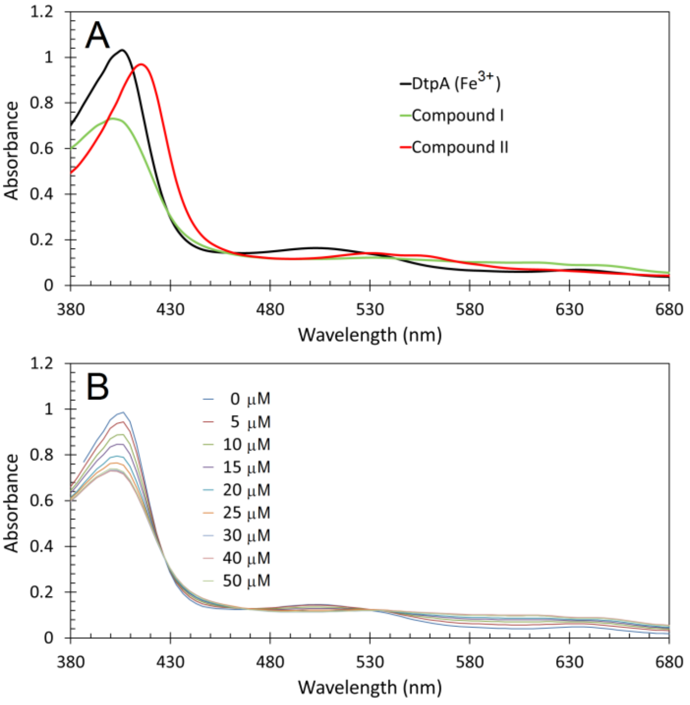


**Figure S2.** A Singular Value Decomposition[9] (SVD) and global analysis of DtpA spectra changing on addition of iron in the presence of apoBFR. **A** – The three basic components spectra output of the analysis of the time dependent spectra of 18 µM DtpA in an oxygenated (at ambient oxygen) solution of 0.5 µM apoBFR after addition of 50 µM iron (100 $Fe^{2+}$/24mer). The spectra are consistent with the model DtpA ($Fe^{3+}$) → Compound I → Compound II. **B** - Formation of Compound I in the oxygenated at ambient conditions system of 18 µM DtpA and 0.5 µM apoBFR as it is mixed with different concentrations of $Fe^{2+}$ (indicated). The spectra are outputs of the analysis of the whole set of spectra obtained in the mixing stopped-flow experiments.

Figure S3

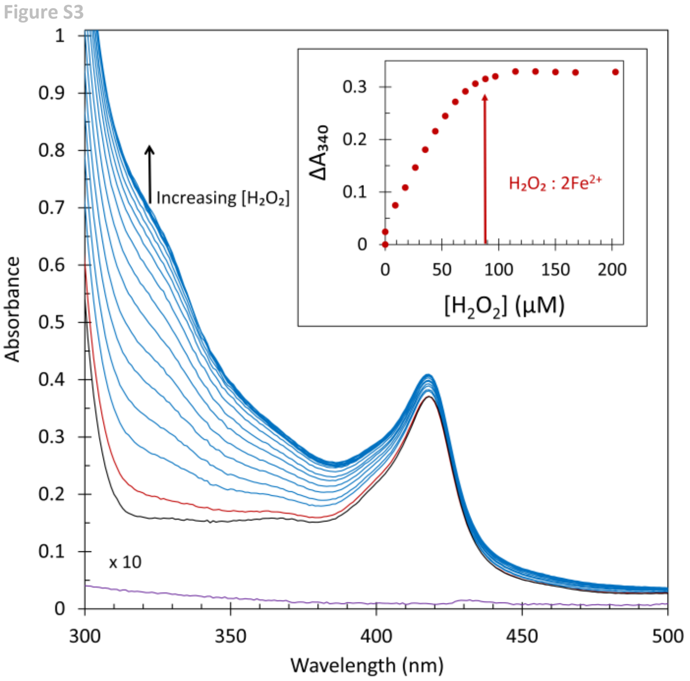


**Figure S3.** UV-vis spectra of the anaerobic EcBFR-$Fe^{2+}$ complex titrated with hydrogen peroxide (compare to the $O_2$ titration experiment reported in Figure S1). Apo-EcBFR (4.24 µM) was incubated anaerobically with 190 µM $Fe^{2+}$ (which is slightly less than required for complete occupancy of all FCs - 4.24 µM x 24 subunits per EcBFR x 2 iron binding sites per FC = 203.5 µM) and then 1.95 mM $H_2O_2$ in an anaerobic MES buffer (100 mM, pH 6.5) was titrated into the mixture. The spectrum of the apo-EcBFR is given in black. After $Fe^{2+}$ was added, about 7% of iron was oxidised by residual $O_2$ (red spectrum, NB two data points for the [$H_2O_2$] = 0 in the *inset*). The blue spectra represent subsequent additions of aliquots of $H_2O_2$ resulting in the range of final $H_2O_2$ concentrations from 6.4 µM to 114.3 µM, reacting with the remaining 93% $Fe^{2+}$ (176.7 µM). A 200 µM $Fe^{2+}$ spectrum multiplied by a factor of 10 is shown in purple at the bottom of the figure. The *inset* shows the oxidation of iron as measured by absorbance increase at 340 nm, as function of $H_2O_2$ concentration, corrected for dilution of the protein. The red arrow indicates the expected $H_2O_2$ concentration, 88.35 µM, corresponding to the 2:1 stoichiometry of iron oxidation by peroxide (176.7 µM $Fe^{2+}$: 88.35 µM $H_2O_2$).

Figure S4

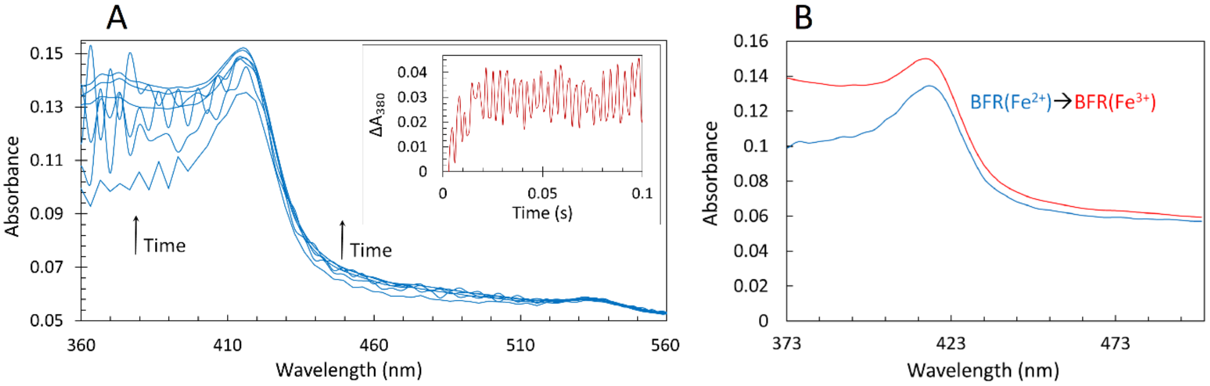


**Figure S4.** The PDA UV-vis spectra of apo-EcBfr pre-loaded anaerobically with $Fe^{2+}$ after $H_2O_2$ in 100 mM MES buffer (pH 6.5) was added. The concentrations in the final mixtures were: [apo-EcBfr] = 1 µM, [$Fe^{2+}$] = 48 µM and [$H_2O_2$] = 40 µM. **A -** The selected spectra shown correspond to the following times: 0.0012, 0.05, 0.01, 0.02, 4.09, 8.07 and 20.52 s. The noise is the consequence of the very fast spectral change - they change markedly over the PDA's intrinsic time of spectrum capture. The *inset* shows the time dependence, at a 3 ms time resolution, of the absorbance increase at 380 nm associated with $Fe^{2+}$ to $Fe^{3+}$ oxidation. NB: Just a **1.67**-fold stoichiometric excess of $H_2O_2$ over iron ((40 µM x 2)/ 48 µM) results in the reaction being completed in about **20 ms**. This may be contrasted with the ~**3 s** necessary for completion of iron oxidation in EcBfr when oxygen is used as an oxidant at a much higher stoichiometric excess of **50** = (600 µM x 4) / 48 µM (Figure 2A). **B -** An SVD analysis output of the whole PDA spectra set obtained from the stopped-flow experiments. The two spectra correspond to the initial spectrum of BFR-$Fe^{2+}$ and final spectrum of BFR-$Fe^{3+}$.

Figure S5

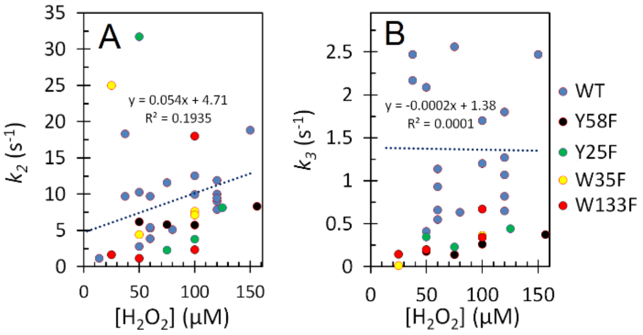


**Figure S5.** With reference to Figure 5 – the absorbance changes at 340 nm during iron oxidation in the FCs of EcBfr by hydrogen peroxide have been monitored by a PM (see examples of the oxidation kinetics in Figure 5A) and fitted with Equation 3. The rate constant $k_1$ change on [$H_2O_2$] is given in Figure 5B and here are the dependences of $k_2$ (**A**) and $k_3$ (**B**). The final concentrations of EcBfr and $Fe^{2+}$ were 1 µM and 48 µM, respectively. The straight dotted lines are linear fits of the two arrays of data.

Figure S6

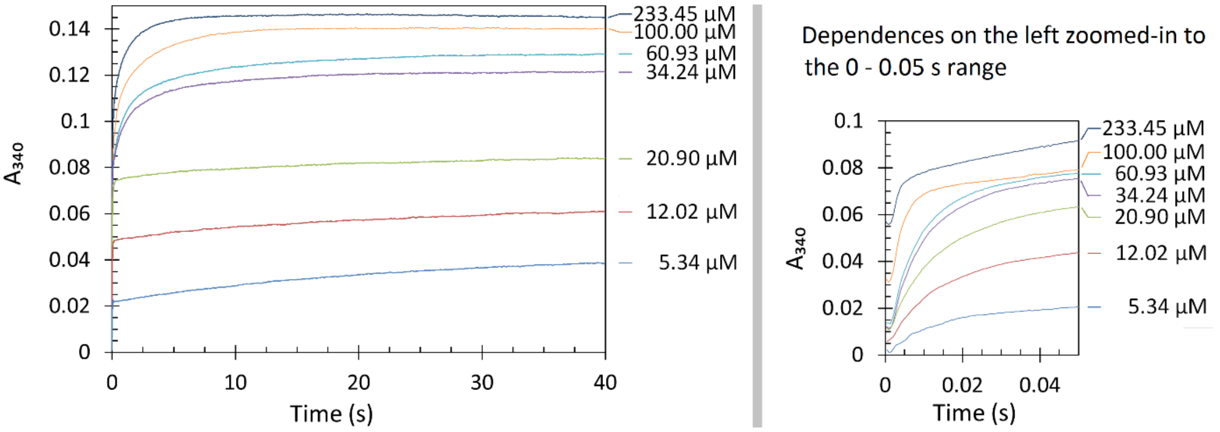


**Figure S6.** The absorbance increase at 340 nm as measured by the PM on addition of buffer with different $H_2O_2$ concentrations to apo-EcBfr anaerobically loaded with $Fe^{2+}$ (the final concentrations in the mixture were [apo-EcBfr] = 2 µM, [$Fe^{2+}$] = 96 µM and [$H_2O_2$] as indicated on the graph). The panel on the right shows the initial 50 ms of the kinetic curves (the fast phase) in greater detail. The traces shown are corrected (shifted up) for the absorbance lost during the dead time (1.5 ms) of the stopped-flow apparatus, the values calculated for each trace from the pseudo-first-order rate constant $k_1$ (the fastest process) obtained from the fits of the traces with Equation 3. The amplitude of the fast phase increases proportionally to $H_2O_2$ concentration – until the point at which the concentration becomes higher than necessary for oxidation of all ferrous iron in the FCs.

Figure S7

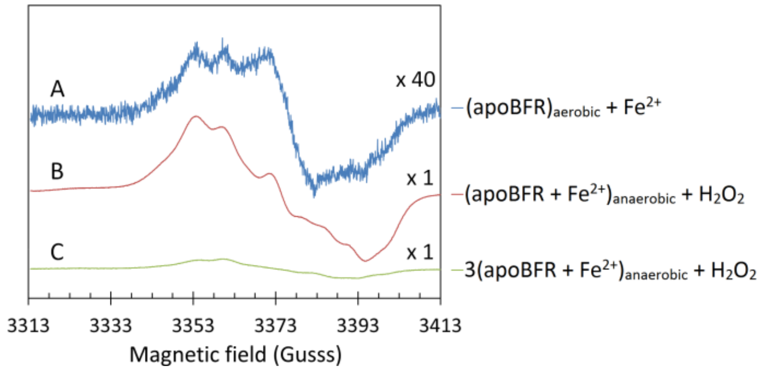


**Figure S7.** EPR spectra of free radicals formed on EcBfr during iron oxidation in the FCs – by $O_2$ and by $H_2O_2$. **A** - ((8.33 µM apo-EcBfr)$_{\text{at ambient oxygen}}$ + 400 µM $Fe^{2+}$), that is 48 Fe/24mer, frozen 17 s after mixing. **B** – ((8.33 µM apo-EcBfr + 400 µM $Fe^{2+}$)$_{\text{anaerobic}}$ + 250 µM $H_2O_2$), frozen 10 s after mixing. **C** – ((25 µM apo-EcBfr + 1200 µM $Fe^{2+}$)$_{\text{anaerobic}}$, that is still 48 Fe/24mer, + 250 µM $H_2O_2$), frozen 10 s after mixing. The samples were frozen by the slow-freeze method. Relative magnifications of the spectra are indicated by the x symbol. The EPR spectra were taken at 23 K.

Figure S8

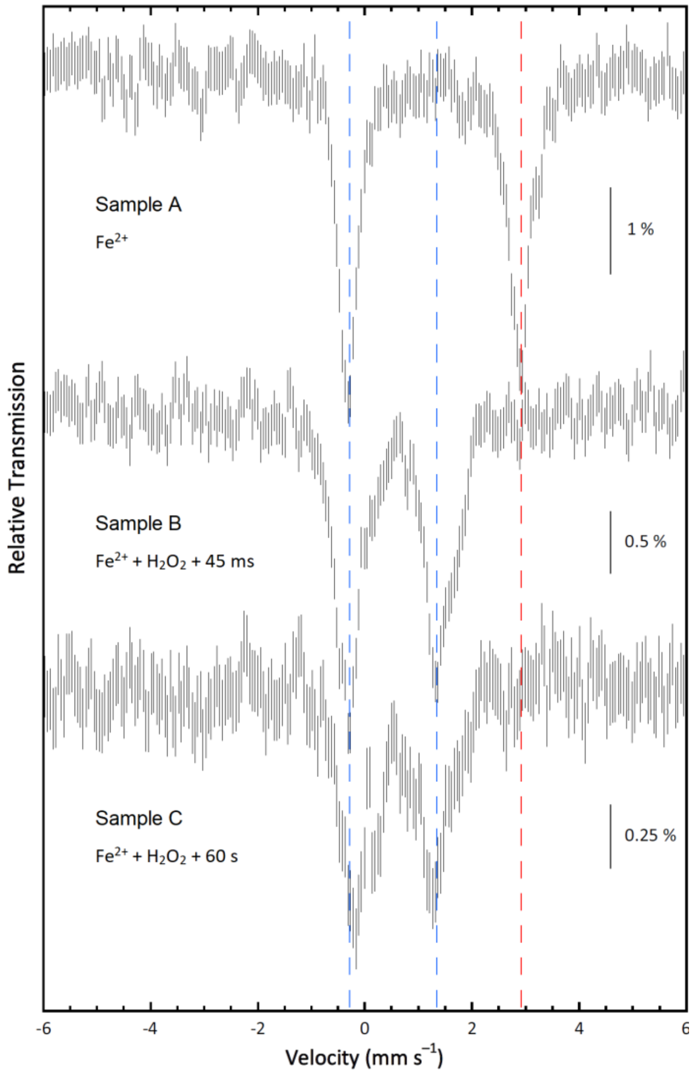


**Figure S8.** The 4.3 K Mössbauer spectra of RFQ samples A, B and C (ferrous loaded EcBfr, 45 ms after $H_2O_2$ addition and 1 min after the addition, respectively). The spectra were recorded using a 60 mT external magnetic field applied parallel to the $\gamma$-rays.

Figure S9

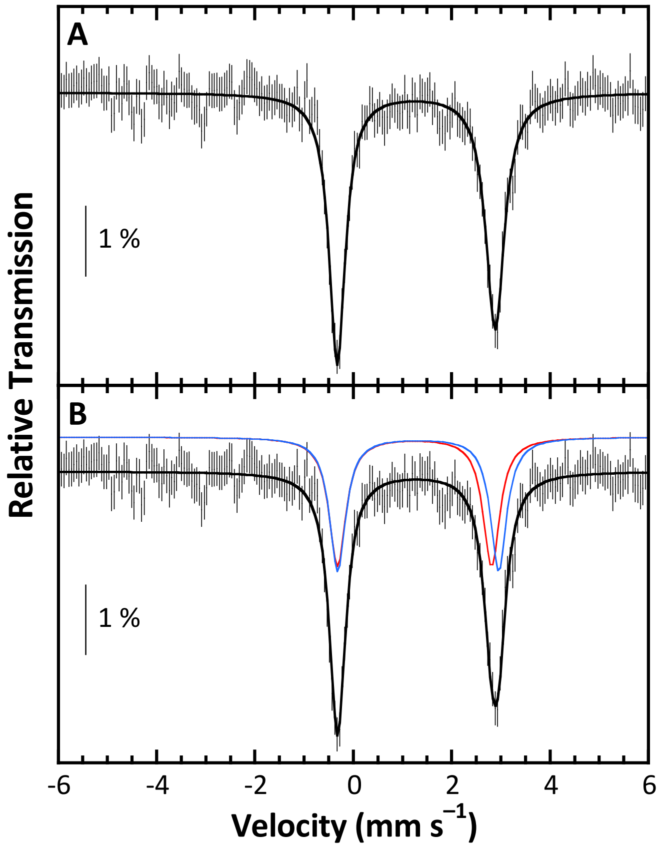


**Figure S9.** Experimental Mössbauer spectrum (hatched bars) of Sample A (41.7 µM apoBFR + 48 $^{57}Fe^{2+}$/24mer, anaerobic) recorded at 4.2 K under a 60 mT external magnetic field applied parallel to the γ-beam and two versions of its simulation (black solid lines): **A** - as a single species with the two lines of the doublet having the same area but two different linewidths; **B** - as a superposition, at equal contribution, of two species with equal widths of the lower and higher velocity components but different between the two doublets (coloured traces). Parameters of the doublets are listed in Table S2. The nuclear parameters determined within the two identical iron sites hypothesis (a single doublet δ = 1.28 mm $s^{-1}$, $\Delta E_Q$ = 3.21 mm $s^{-1}$, see Table S2) are close to the average values reported for *E. coli* RNR (δ = 1.26 mm $s^{-1}$, $\Delta E_Q$ = 3.13 mm $s^{-1}$).[10] When the spectrum was simulated assuming two different iron sites, the parameters ($\delta_1$ = 1.24 mm $s^{-1}$, $\Delta E_{Q1}$ = 3.13 mm $s^{-1}$; $\delta_2$ = 1.32 mm $s^{-1}$, $\Delta E_{Q2}$ = 3.27 mm $s^{-1}$, see Table S2) were similar to those obtained for the ferroxidase centre in the ferrous state of the bacterioferritin from *Desulfovibrio vulgaris*[11] ($\delta_1$ = 1.22 mm $s^{-1}$, $\Delta E_{Q1}$ = 3.20 mm $s^{-1}$, $\delta_2$ = 1.46 mm $s^{-1}$, $\Delta E_{Q2}$ = 3.24 mm $s^{-1}$). Two distinct ferrous sites with similar parameters have been identified in soluble methane monooxygenase hydroxylase from *Methylosinus trichosporium* OB3b ($\delta_1$ = 1.26 mm $s^{-1}$, $\Delta E_{Q1}$ = 3.22 mm $s^{-1}$; $\delta_2$ = 1.35 mm $s^{-1}$, $\Delta E_{Q2}$ = 2.37 mm $s^{-1}$.[12]

Figure S10

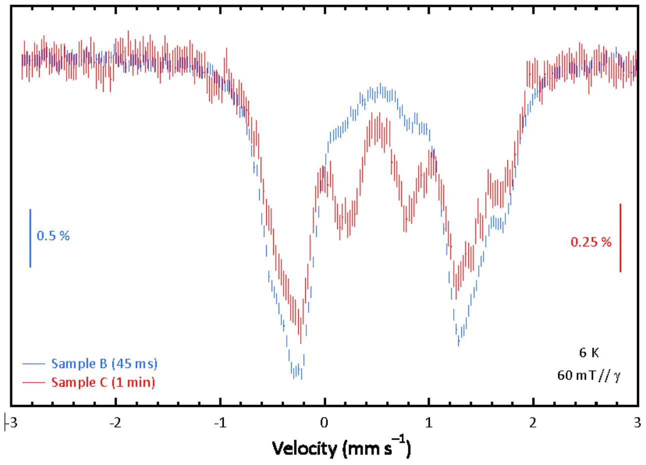


**Figure S10.** Mössbauer spectra of BFR samples treated with $H_2O_2$ and frozen 45 ms (blue) and 60 s (red) thereafter. The spectra were measured at 6 K with a 60 mT external magnetic field applied along the γ-beam. The spectra are normalised to a common total integral assuming the total concentration of $^{57}Fe$ is the same in the two samples.

Figure S11

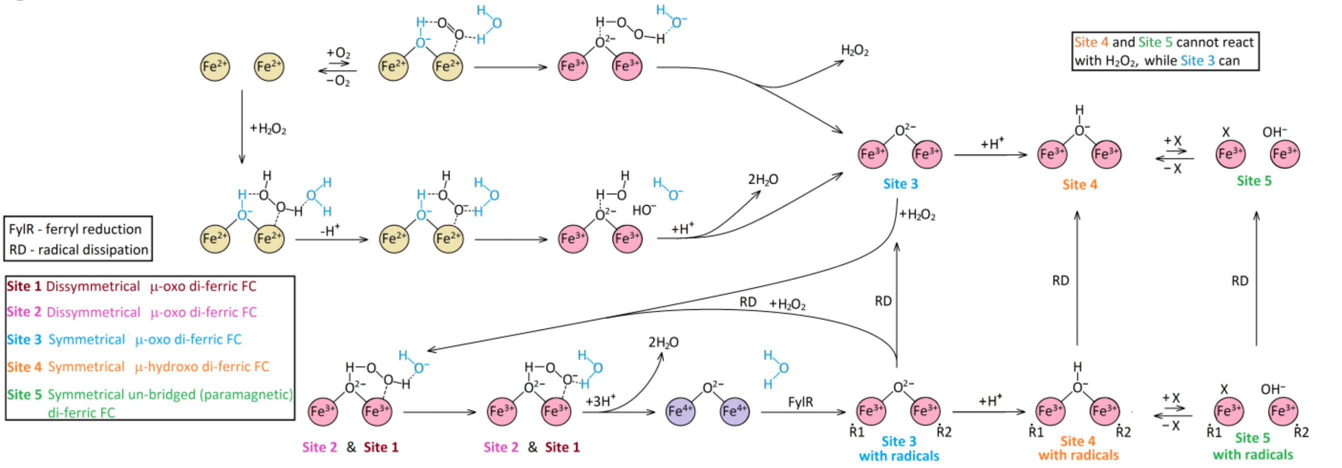


**Figure S11.** Iron oxidation at the di-iron FC of EcBfr – with $O_2$ and with intermediately formed $H_2O_2$. The diagram is a more detailed version of Figure 8, with some assumptions and references to literature data.

Specifically, the diagram shows hypothetical water molecules and hydroxyl groups (shown in blue colour) that might participate in coordination of the oxidants and in proton exchange. $O_2$ binds reversibly to one of the two iron ions of the di-ferrous FC, relatively rapidly but weakly as indicated by $K_D$ = 8.23 x $10^{-4}$ M (at 25ºC, Figure 3 and Table S1). This makes the population of the $2Fe^{2+}$-$O_2$, and hence the observed rate of iron oxidation by $O_2$, low (~0.9 $s^{-1}$ at 25°C and 260 μM $O_2$, see Figure 3B). This rate, although consistent with literature values for EcBfr with low haem content,[13] is notably lower than the pseudo-first-order values reported for iron oxidation by oxygen in other di-iron enzymes, for example, 26 $s^{-1}$ in ToMOHred [14] or 22 $s^{-1}$ (at 700 μM [$O_2$]) in MMOH.[15] Second order $O_2$ binding by the di-ferrous centre of Hr was reported with a $k_{on}$ ~$10^7$ $M^{-1}s^{-1}$.[16] An estimate of the pseudo-first-order rate constant for $O_2$ binding to Hr, calculated from the second order value of 2.6 x $10^7$ $M^{-1}$ $s^{-1}$ [16a] and an $O_2$ concentration of 260 μM, yields $k_1$ = 6700 $s^{-1}$, three orders of magnitude greater than $k_{max}$ observed here.

The rate of iron oxidation by $H_2O_2$, on the other hand, is comparable with those of dedicated peroxidases, and for a range of $H_2O_2$ concentrations similar to ambient $O_2$ concentrations is ~3 orders of magnitude higher than pseudo-first-order rate constant of oxidation by $O_2$ (see Figure 5B vs Figure 3B).

The diagram also provides tentative assignments of the five ferric sites identified by the Mössbauer spectroscopy. Site 1 - one of the two ferric atoms of the 'dissymmetrical' di-ferric μ-oxo-bridged FC. Site 2 – the other of the two ferric atoms of the 'dissymmetrical' di-ferric μ-oxo-bridged FC. Sites 1 and 2 are antiferromagnetically coupled to an overall diamagnetic di-ferric state. Site 3 - two identical ferric atoms in the 'symmetrical' μ-oxo-bridged FC, also yielding an overall diamagnetic state FC. Site 4 - two identical ferric atoms in a 'symmetrical' μ- hydroxo-bridged FC, also diamagnetic. Site 5 - two identical FC ferric atoms un-bridged and therefore paramagnetic. We propose that Site 5 is formed from its precursor, Site 4, by accepting a second ligand X to already formed μ-hydroxo di-ferric FC (Figure 8) which breaks the antiferromagnetic coupling and creates two paramagnetic high spin ferric species. Since the paramagnetism is not manifested as appearance of distinct two new species, it is possible that ligand X is also a hydroxide $OH^-$.

## Supporting tables

Table S1

**Table S1.** The maximal rate constants $k_{obs}^{max}$ of iron oxidation in the FC of EcBfr by $O_2$ and the $O_2$ dissociation constants $K_D$ as determined at two different temperatures (with reference to Figure 3 and Equation 2).[a]

| | 25°C | 10°C |
|---|---|---|
| $k_{obs}^{max}$ | 3.68 $s^{-1}$ | 0.87 $s^{-1}$ |
| $K_D$ | 8.23 x $10^{-4}$ M | 2.45 x $10^{-4}$ M |

[a] The values of $k_{obs}^{max}$ and $K_D$ at 25°C and 10°C were used to estimate the activation energy and the standard enthalpy of the equilibrium between $O_2$ and the FC: $\Delta E_a$ = 67.4 kJ $mol^{-1}$ and ΔH⊖ = 56.6 kJ $mol^{-1}$.

Table S2

**Table S2.** The parameters used to simulate the experimental spectrum shown in Figure S9.

| | Site | $\delta$ (mm $s^{-1}$) | $\Delta E_Q$ (mm $s^{-1}$) | $\Gamma$ (mm $s^{-1}$)[a] |
|---|---|---|---|---|
| **One doublet** | | 1.28 | 3.21 | 0.40 / 0.46 |
| **Two doublets at a 1:1 ratio** | red | 1.24 | 3.13 | 0.41 |
| | blue | 1.32 | 3.27 | 0.40 |

[a] Full-width at half-maximum

Table S3

**Table S3.** Parameters of the theoretical traces used in simulation of the Mössbauer spectra in Figure 7A and Figure 7B (colour-coordinated with Figure 7 and Figure S11).

| | δ (mm $s^{-1}$) | $\Delta E_Q$ (mm $s^{-1}$) | η | $\Gamma_{FWHM, 60 mT}$ (mm $s^{-1}$)[a] | $\Gamma_{FWHM, 7 T}$ (mm $s^{-1}$)[a] | Contribution in Sample B (%) | Contribution in Sample C (%) |
|---|---|---|---|---|---|---|---|
| Site 1 (S = 0) | 0.59 | -2.24 | 0.07 | 0.27 | 0.42 | 27 | 11 |
| Site 2 (S = 0) | 0.55 | -1.79 | 0.57 | 0.27 | 0.42 | 29 | 16 |
| Site 3 (S = 0) | 0.52 | -1.46 | 0.61 | 0.27 | 0.42 | 46 | 27 |
| Site 4 (S = 0) | 0.50 | 0.60 | 0.97 | 0.27 | 0.42 | 5 | 23 |
| Site 5[b] (S = 5/2) | 0.48 | 0.24 | 0.22 | 0.38 | 0.31 | 0 | 27 |
| Uncertainty | ±0.02 | ±0.05 | ±0.05 | ±0.03 | ±0.03 | ±5 | ±5 |

[a] A common value was assumed for the S=0 species
[b] Additional parameters determined for these sub-spectra: $D$=–0.66 $cm^{-1}$, $E/D$ = 0.25, $a_k/(g_n\mu_n)$ = –21.7, –22.7, –22.3 T ($k$=$x,y,z$)

## Abbreviations and annotations used

Bfr, bacterioferritin - bacterial ferritin that has a haem at the interface of subunits in a dimer;
BfrB, *Pseudomonas aeruginosa* Bfr;
Dps, mini-ferritin, DNA-binding protein from starved cells;
EcBfr, *Escherichia coli* Bfr;
EPR, Electron Paramagnetic Resonance;
FC, ferroxidase centre;
Ftn, bacterial ferritin made of subunits similar to animal H-chains;
FtnA, *Escherichia coli* Ftn ferritin responsible for iron homeostasis;
Hr, haemerythrin;
IS, inner surface (iron binding site);
PDA, photodiode array;
RFQ, Rapid Freeze-Quenching;
RNR, ribonucleotide reductase;
SVD, Singular Value Decomposition;
UV-vis, ultraviolet-visible (spectrophotometry);
WT, wild type.

## References

[1] a) J. M. Bradley, D. A. Svistunenko, T. L. Lawson, A. M. Hemmings, G. R. Moore, N. E. Le Brun, *Angew. Chem. Int. Ed. Engl.* **2015**, *54*, 14763–14767; b) T. L. Lawson, A. Crow, A. Lewin, S. Yasmin, G. R. Moore, N. E. Le Brun, *Biochemistry* **2009**, *48*, 9031-9039.
[2] E. R. Bauminger, P. M. Harrison, D. Hechel, I. Nowik, A. Treffry, *Biochim. Biophys. Acta* **1991**, *1118*, 48-58.
[3] N. E. Le Brun, M. T. Wilson, S. C. Andrews, J. R. Guest, P. M. Harrison, A. J. Thomson, G. R. Moore, *FEBS Lett.* **1993**, *333*, 197-202.
[4] a) A. Crow, T. L. Lawson, A. Lewin, G. R. Moore, N. E. Le Brun, *J. Am. Chem. Soc.* **2009**, *131*, 6808-6813; b) X. Yang, N. E. Le Brun, A. J. Thomson, G. R. Moore, N. D. Chasteen, *Biochemistry* **2000**, *39*, 4915-4923.
[5] M. R. Cheesman, N. E. le Brun, F. H. Kadir, A. J. Thomson, G. R. Moore, S. C. Andrews, J. R. Guest, P. M. Harrison, J. M. Smith, S. J. Yewdall, *Biochem. J.* **1993**, *292 ( Pt 1)*, 47-56.
[6] J. K. Gundersen, N. B. Ramsing, R. N. Glud, *Limnol. Oceanogr.* **1998**, *43*, 1932-1937.
[7] M. K. Thompson, S. Franzen, R. A. Ghiladi, B. J. Reeder, D. A. Svistunenko, *J. Am. Chem. Soc.* **2010**, *132*, 17501-17510.
[8] a) M. Carboni, M. Clemancey, F. Molton, J. Pecaut, C. Lebrun, L. Dubois, G. Blondin, J. M. Latour, *Inorg. Chem.* **2012**, *51*, 10447-10460; b) M. Carboni, M. Clemancey, F. Molton, J. Pecaut, C. Lebrun, L. Dubois, G. Blondin, J. M. Latour, *Inorg. Chem.* **2012**, *51*, 12053-12053; c) C. Charavay, S. Segard, F. Edon, M. Clémancey, G. Blondin, CEA/iRTSV, CNRS, Université Grenoble Alpes., Grenoble, **2019**.
[9] G. H. Golub, C. F. Van Loan, *Matrix Computations*, 2nd ed. ed., Johns Hopkins University Press, Baltimore, **1989**.
[10] J. B. Lynch, C. Juarez-Garcia, E. Munck, L. Que, Jr., *J. Biol. Chem.* **1989**, *264*, 8091-8096.
[11] C. G. Timoteo, M. Guilherme, D. Penas, F. Folgosa, P. Tavares, A. S. Pereira, *Biochem. J.* **2012**, *446*, 125-133.
[12] a) B. G. Fox, M. P. Hendrich, K. K. Surerus, K. K. Andenson, W. A. Froland, J. D. Lipscomb, E. Munck, *J. Am. Chem. Soc.* **1993**, *115*, 3688-3701; b) R. Banerjee, K. K. Meier, E. Munck, J. D. Lipscomb, *Biochemistry* **2013**, *52*, 4331-4342.
[13] S. G. Wong, R. Abdulqadir, N. E. Le Brun, G. R. Moore, A. G. Mauk, *Biochem. J.* **2012**, *444*, 553-560.
[14] L. J. Murray, S. G. Naik, D. O. Ortillo, R. Garcia-Serres, J. K. Lee, B. H. Huynh, S. J. Lippard, *J. Am. Chem. Soc.* **2007**, *129*, 14500-14510.
[15] S. K. Lee, J. C. Nesheim, J. D. Lipscomb, *J. Biol. Chem.* **1993**, *268*, 21569-21577.
[16] a) G. Bates, M. Brunori, G. Amiconi, E. Antonini, J. Wyman, *Biochemistry* **1968**, *7*, 3016-3020; b) D. J. de Waal, R. G. Wilkins, *J. Biol. Chem.* **1976**, *251*, 2339-2343.

## Author Contributions

J. Pullin - performed the experiments and data analysis, wrote original draft - equal
M. Wilson –performed the experiments and data analysis, wrote parts of the original draft, edited the final version– lead
M. Clémancey - performed the experiments and data analysis -- equal
G. Blondin - performed the experiments and data analysis - equal
J. Bradley - performed the experiments, wrote parts of the initial draft - equal
G. Moore – wrote parts of the original draft, edited the manuscript - equal
N. Le Brun – funding acquisition, edited the manuscript – equal
M. Lučić – performed the experiments, data analysis - equal
J. Worrall - edited the manuscript - equal
D. Svistunenko - funding acquisition, performed the experiments and data analysis, wrote the final version of the manuscript – lead
